# Identifying hub genes, key pathways and key immune-related genes in Peyronie’s disease by integrated bioinformatic analysis

**DOI:** 10.3389/fphar.2022.1019358

**Published:** 2022-12-06

**Authors:** Yuanshan Cui, Lili Chen, Xiaofeng Wang, Luxin Yu, Jitao Wu

**Affiliations:** ^1^ Department of Urology, The Affiliated Yantai Yuhuangding Hospital, Qingdao University, Yantai, China; ^2^ Department of Reproductive Medicine, The Affiliated Yantai Yuhuangding Hospital, Qingdao University, Yantai, China

**Keywords:** immune, Peyronie’s disease, bioinformatics, gene expression omnibus, hub genes

## Abstract

Scarring diseases, such as Peyronie’s disease (PD), usually lead to disorders in the immune system. Previous studies suggested that the PD process was regulated by immune signaling. However, the pathogenetic mechanism remains incompletely characterized. This article used bioinformatic approaches to identify hub genes, key pathways and key immune-related genes that play essential roles in PD pathogenesis. Two Gene Expression Omnibus (GEO) datasets, GSE126005 and GSE146500, were used to analyse the transcriptional profiling in both PD and normal samples. R software was applied to examine the difference in the expression of hub genes and key immune-related genes. The candidates for hub genes were further validated through protein–protein interactions (PPIs), gene correlation, and Kyoto Encyclopedia of Genes and Genomes (KEGG) pathway analyses. In addition, candidate miRNA‒mRNA pairs were functionally assessed. A total of 39 candidate genes were identified, the expression levels of which in PD fibroblast cells were different from those in normal cells (16 showed reduced expression in PD and 21 candidates overexpressed in PD). We found that these genes could interact with each other through PPI analysis. According to the functional enrichment analysis, the candidates may regulate some major biological processes, including cytokine‒cytokine receptor interactions and the JAK-STAT signaling pathway. IL6, IL21R, IFNE, CXCL2, EGF, and ANGPTL5 were identified as key immune-related genes. The findings may help understand the role of immunologic contributors in PD, thus shedding light on the development of more effective strategies to prevent and treat this kind of disease.

## Introduction

Peyronie’s disease (PD) is a disease caused by fibrotic plaque developed in the penis tissue, which often leads to curved and painful erections that affect 0.4%–9% of men (Cui et al., 2022). The pathophysiology of PD is commonly considered to be the development of fibrotic plaque in the tunica albuginea (TA) following penis trauma (Schirmann et al., 2022). Profibrotic factors, including excessive reactive oxygen species (ROS), transforming growth factor (TGF) β1 and plasminogen activator inhibitor-1, activate and upregulate the production of collagen (Chung and Yafi, 2022). In addition to reduced elasticity in TA, PD also results in penile deformity and intense pain, which significantly impair intercourse performance and mental health (Krakhotkin et al., 2020). Furthermore, PD leads to many complications that may remarkably reduce the quality of life of males (Milenkovic et al., 2020). Due to the lack of understanding of the pathology, there is currently no conservative or medical treatment for PD (Pyrgidis et al., 2021). Therefore, elucidating the underlying pathogenic mechanisms, especially the molecular signaling processes and transcriptional changes, could help develop clinical strategies to treat PD.

Previous studies have demonstrated the essential role of the immune system during the fibrosis process in PD fibrosis. Microarray assays by Magee et al. (2002) reported that the expression levels of genes related to proinflammatory factors, such as MCP-1, pleiotrophin, and early growth response protein, were elevated in PD patients. The role for T lymphocytes and macrophages was identified by Ralph et al. (2019) 20 years ago by analysing acute-phase PD plaques. The immunohistochemistry (IHC) and single-cell RNA sequencing (scRNA-Seq) analyses carried out by Milenkovic et al. (2021) indicated the existence of an inflammatory reaction in the chronic stage of PD. Inflammation is driven by specialized monocyte-derived cytotoxic T lymphocytes (CTLs) and mucosa-associated invariant T (MAIT) cells, which are activated by vascularization and collagen production. Despite these findings, the understanding of immune cell contents and signaling pathways of PD chronic plaques is still very limited.

In this article, key hub genes were identified based on the Gene Expression Omnibus (GEO) datasets GSE126005 and GSE146500. Then, these genes were subjected to further analyses, including protein–protein interactions (PPI), gene correlation, and Kyoto Encyclopedia of Genes and Genomes (KEGG) pathway analyses. According to the functional enrichment analysis, these genes may play regulatory roles in some major biological processes, including cytokine‒cytokine receptor interactions and the JAK-STAT signaling pathway. Ultimately, the genes IL6, IL21R, IFNE, CXCL2, EGF, and ANGPTL5 were identified to be immune-related. In addition, candidate miRNA‒mRNA pairs were functionally assessed. This study will help improve the understanding of PD pathogenesis, which may facilitate the development of new strategies to treat PD in the clinic.

## Materials and methods

### Microarray data

The GSE126005 and GSE146500 datasets containing transcriptional profiles of PD and normal patient samples were acquired through the GEO website http://www.ncbi.nlm.nih.gov/geo; (Milenkovic et al., 2021; Yin et al., 2021). The expression profiling analysis of GSE126005 was conducted on the GPL18573 high-throughput sequencing platform using Illumina NextSeq 500 (Homo sapiens) Technology. Among the datasets, 6 samples were from normal human fibroblasts (undergoing penile surgery for penile cancer), and 6 samples were taken from PD fibroblast plaques. The expression profiling of GSE146500 was conducted on the GPL16791 platform using Illumina HiSeq 2,500 (Homo sapiens) Technology. The objects contained 4 normal fibroblast samples from undergoing penoplasty for congenital curvature and 4 PD fibroblast samples. Since the database is open-access, no authorization from the local ethics committee was needed.

### Differential expression analysis of key hub genes

The difference in gene expression between PD and normal samples was determined according to the expression profiling of datasets GSE126005 and GSE146500. The probe annotation was applied as instructed, and the batch effect was excluded through principal component analysis (PCA). Key hub genes were primarily identified using the “limma” package of R software. The changes in expression levels were considered to be significantly different when adjusted absolute fold-change >1 and *p* < 0.05. The “heatmap” and “ggplot2” packages of R software were utilized for visualizing the interpretation of the results.

### Protein-protein interaction (PPI) and gene correlation analysis of the key hub genes

PPI analysis was carried out by applying Cytoscape software (version 3.8.1) based on the Search Tool for the Retrieval of Interacting Genes/Proteins (STRING) database (https://string-db.org/). Minimum required interaction score is 0.4. The gene correlations were further identified by Spearman correlation in the “corrplot” package of R software.

### Kyoto Encyclopedia of Genes and Genomes (KEGG) pathway and disease enrichment analysis of the key hub genes

KEGG pathway enrichment analysis was conducted by utilizing the Gene Ontology (GO) plot package of R software. *p*-value less than 0.05 is included. Disease enrichment analysis was implemented by Enrichr (https://maayanlab.cloud/Enrichr/).

### Evaluation of immune cell infiltration

The gene expression matrix data were uploaded to CIBERSORTx. By filtering out samples with *p* < 0.05, we were able to obtain the infiltration matrix of immune cells. Then, the correlation of 16 immune cells was visualized using the “corrplot” package.

### Key immune-related Gene-miRNA analysis

A total of 2,484 genes were identified to be associated with immunity according to Gene Set Enrichment Analysis (GSEA; http://www.gsea-msigdb.org/gsea/index.jsp). The candidate immune-related genes, the expression levels of which were altered in PD, were then further determined using the “limma” package of R software. The miRWalk 2.0 database was applied to analyse key immune-related gene-miRNA interactions.

### Statistical analysis

R software (version 3.6.2) was applied for statistical analysis. Student’s t test was assigned to evaluate the significances for differences. The *p*-value less than 0.05 was considered as statistically significant.

## Results

### Identification of hub genes differentially expressed in PD

**FIGURE 1:**
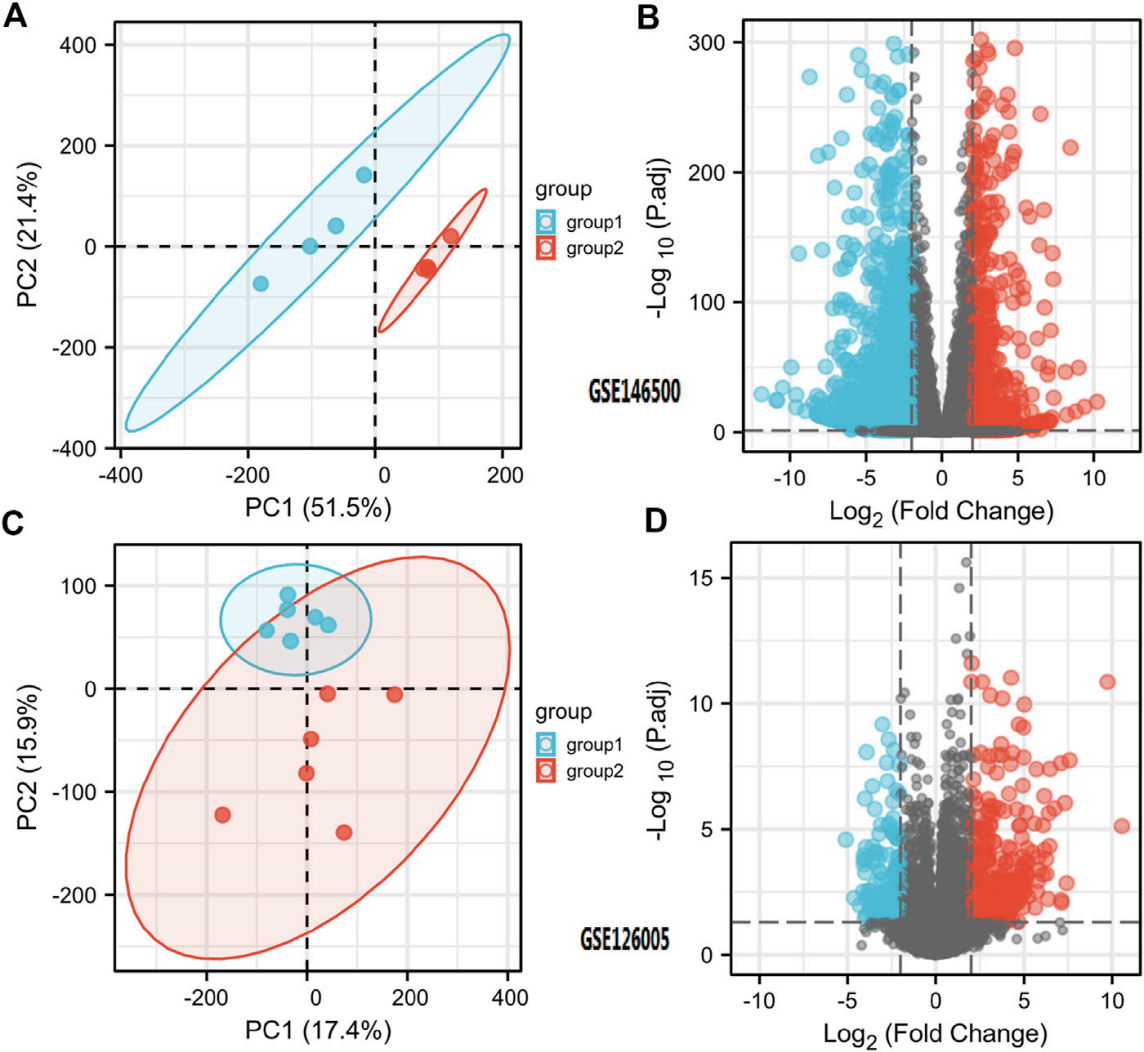
Differentially Expressed Genes in PD and Healthy Samples. **(A)** Principal Components Analysis for GSE146500. **(B)** Volcano Plot of the Differentially Expressed Genes for GSE146500. **(C)** Principal Components Analysis for GSE126005. **(D)** Volcano Plot of the Differentially Expressed Genes for GSE126005. PD = Peyronie’s disease.

**FIGURE 2:**
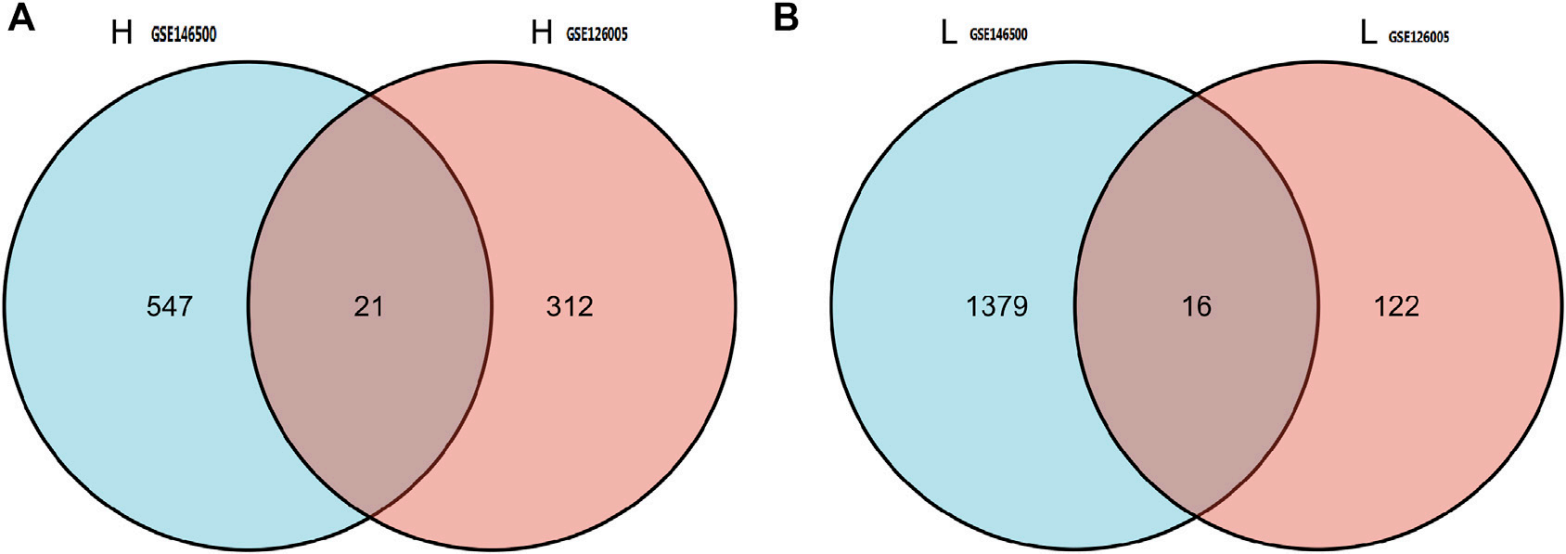
37 of DEGs were shared in these two datasets. Among these genes, 21 were up-regulated, while the other 16 genes were down-regulated. DEGs: differentially expressed genes.

**FIGURE 3:**
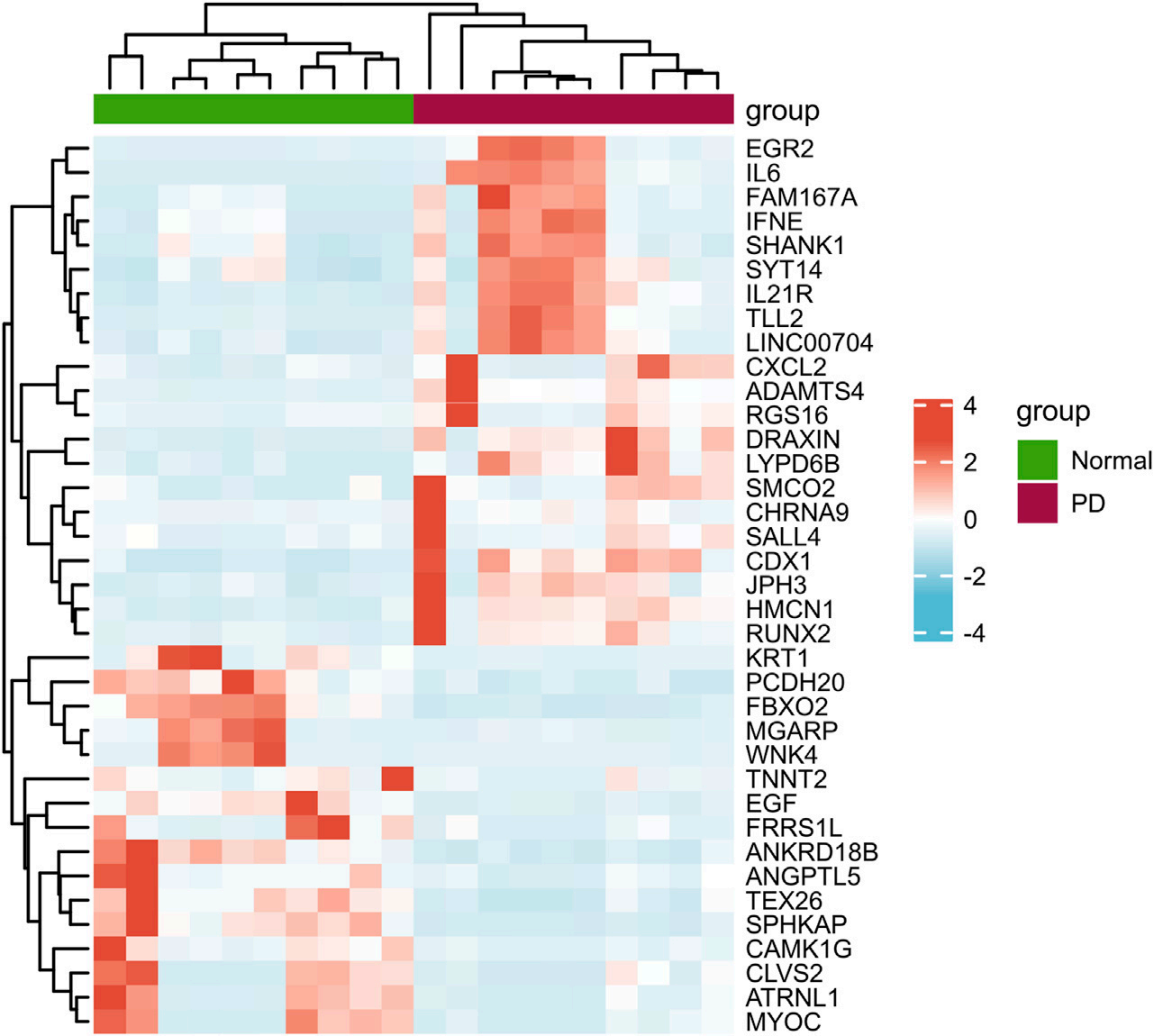
Heatmap of the 37 Differentially Expressed Genes in PD and Healthy Samples. PD = Peyronie’s disease.

**FIGURE 4:**
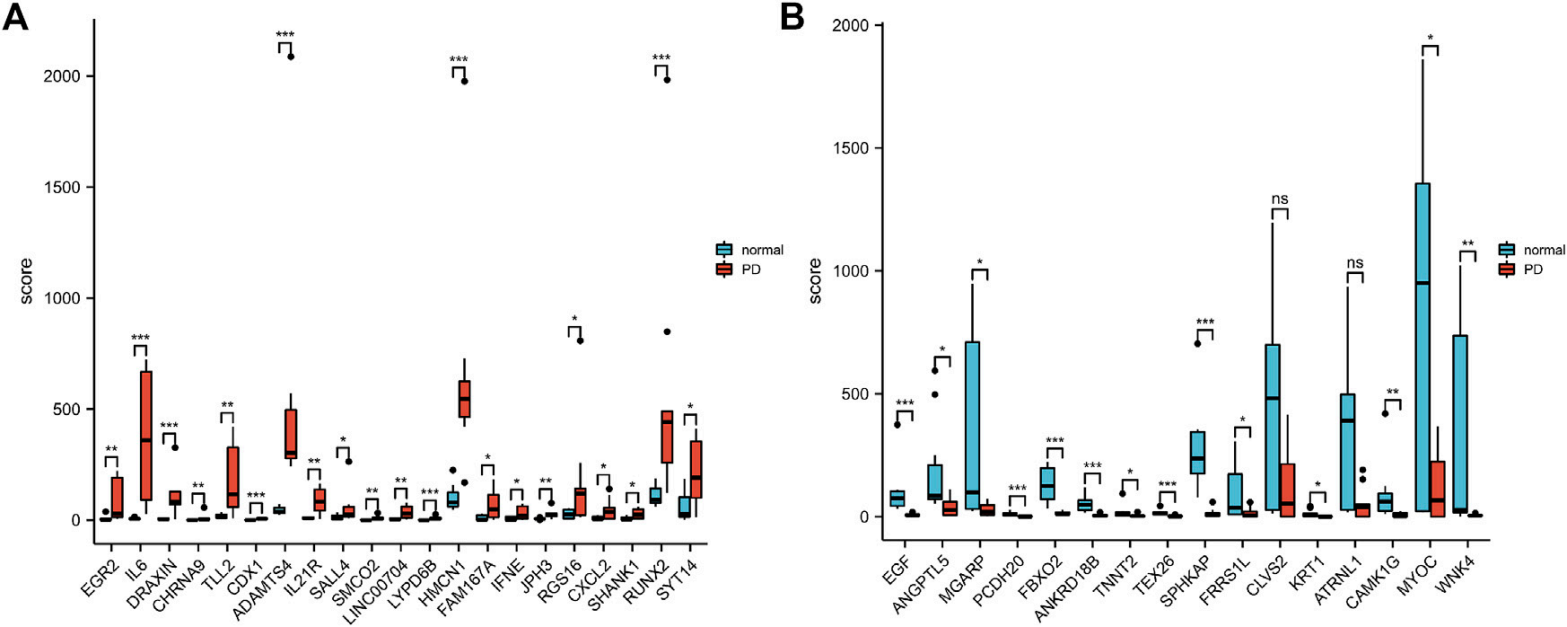
The Boxplot of 37 Differentially Expressed Genes in PD and Healthy Samples. **(A)** The Boxplot of Top 21 Differentially Expressed Genes in PD and Healthy Samples. **(B)** The Boxplot of Last 16 Differentially Expressed Genes in PD and Healthy Samples. PD = Peyronie’s disease.

According to the PCA results, the repetitiveness of intragroup data in GSE146500 and GSE126005 passed the validity check (Figures 1A,C). The profiling for the expression of fibroblasts from chronic phase of PD and normal penile tunica albuginea was analysed and compared using R software. Genes that showed different expression levels between these two groups were selected and presented in a volcano plot (Figures 1B,D). A total of 1963 genes (568 upregulated and 1,395 downregulated) were found to be differentially expressed in GSE146500 and 471 (333 upregulated and 138 downregulated) in GSE126005. The *p*-value was <0.05, and the fold change cut-off was 1.5. Further analysis showed that 37 of these differentially expressed genes (DEGs) were shared in these two datasets (Figure 2). Among these genes, 21 were upregulated, while the other 16 genes were downregulated. Next, we created a heatmap using R software, and the 37 candidate genes are plotted in Figure 3. The box plots also allowed the visualized comparison of the differential expression levels in PD among the 37 candidate genes (Figure 4). The top five hub genes with elevated expression in PD were EGR2, IL6, DRAXIN, CHRNA9, and TLL2, and the top five hub genes suppressed in PD were KRT1, ATRNL1, CAMK1G, MYOC, and WNK4.

### PPI and correlation analysis of the candidate genes

**FIGURE 5:**
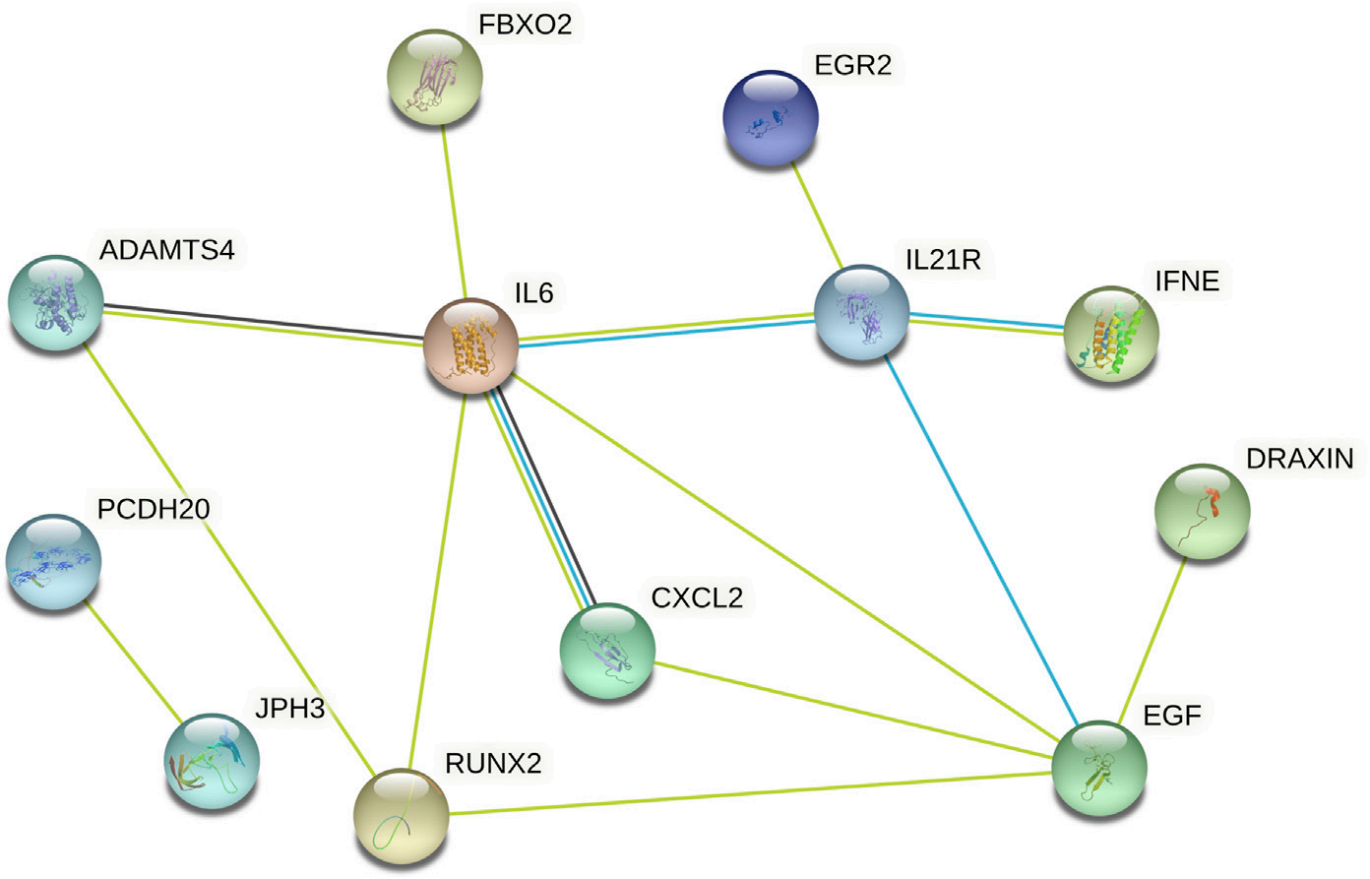
The PPI Among 37 Differentially Expressed Genes. PPI = protein-protein interactions.

**FIGURE 6:**
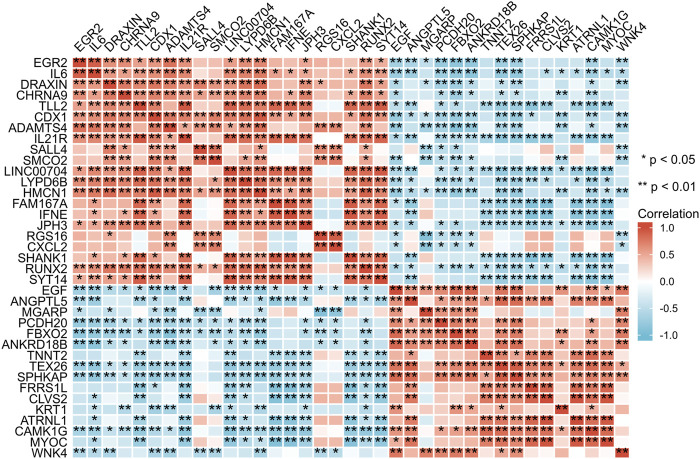
Spearman correlation analysis of the 37 differentially expressed genes.

Subsequently, data from STRING were analysed using Cytoscape software to construct the PPI network. The results indicated that there are protein‒protein interactions among these candidate genes, as shown in Figure 5. The coregulated expression of the candidate genes was assessed through gene correlation analysis (Figure 6).

### Functional enrichment analyses of candidate genes

**FIGURE 7:**
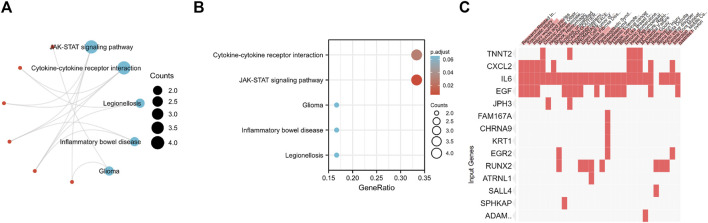
**(A,B)** KEGG enrichment analysis of 37 Differentially Expressed Genes. **(C)** Disease enrichment analysis using Enrichr showed that the top genes are enriched in various disorders including Research-Related Injuries, Traumatic injury, Injury wounds, Wounds and Injuries, Chronic Airflow Obstruction, Headache associated with sexual activity, Shock and Hemorrhagic, Scoliosis and unspecified, AICARDI-GOUTIERES SYNDROME 1 and Hyperlipoproteinemia Type IIa and so on.

To investigate the potential functions of the 37 candidate genes, we conducted KEGG enrichment analysis. According to the KEGG pathway analysis, these genes played essential roles in the regulation of cytokine‒cytokine receptor interactions and the JAK-STAT signaling pathway (Figures 7A,B). Disease enrichment analysis using Enrichr showed that the top genes (TNNT2, CXCL2, IL6, EGF, JPH3, FAM167A, CHRNA9, KRT1, EGR2, RUNX2, ATRNL1, SALL4, SPHKAP, and ADAMTS4) were enriched in various disorders, including research-related injuries, traumatic injury, injury wounds, wounds and injuries, chronic airflow obstruction, headache associated with sexual activity, shock and hemorrhagic, scoliosis and unspecified, AICARDI-GOUTIERES SYNDROME 1 and hyperlipoproteinemia type IIa (Figure 7C).

### Immune cell infiltration

**FIGURE 8:**
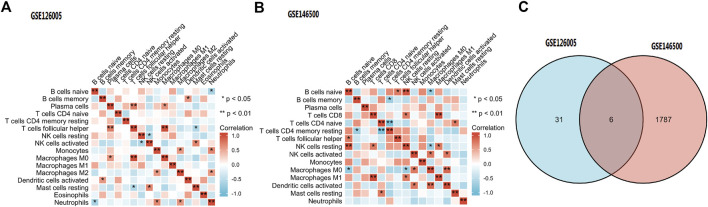
**(A,B)** Immune Cell Infiltration. **(C)** According to the selection criteria (absolute fold-change>1, adjusted *p* < 0.05), 6 key immune-related genes which showed altered expression in PD were distinguished. PD = Peyronie’s disease.

Among the 16 types of immune cells, naive B cells, resting NK cells and follicular helper T cells showed a significant positive correlation according to the correlation heatmap. B naive cells, M0 macrophages and neutrophils had a significant negative correlation. T follicular helper cells, plasma cells and M0 macrophages had a significant positive correlation. CD8 T cells had a significant positive correlation with M1 macrophages (Figures 8A,B).

### Key immune-related gene and gene-miRNA analysis

**FIGURE 9:**
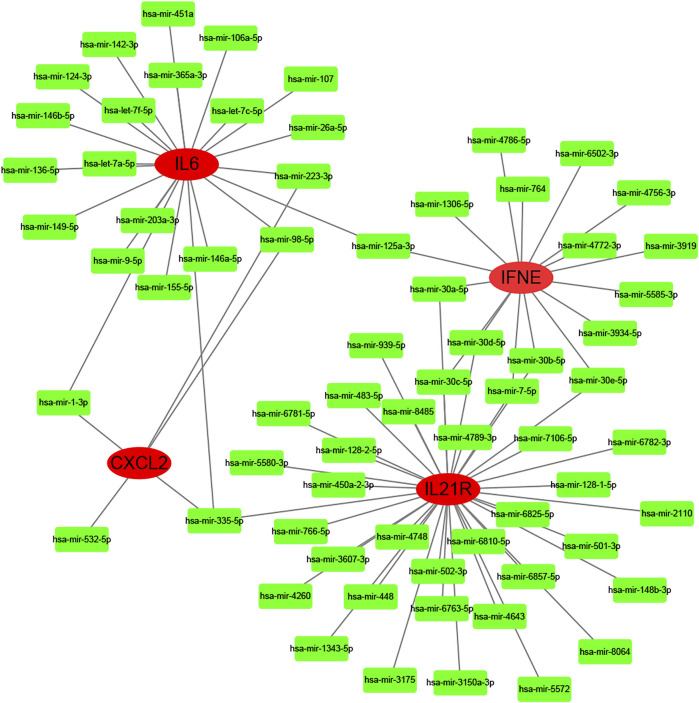
The interactions among the 4 core genes and their target miRNAs.

According to the selection criteria (absolute fold-change>1, adjusted *p* < 0.05), 6 key immune-related genes that showed altered expression in PD were distinguished (Figure 8C). There were IL6, IL21R, IFNE, CXCL2, EGF, and ANGPTL5. Interestingly, the first five genes were the core genes in the PPI network. Then, the interaction between the core genes and miRNAs was analysed. Hsa-miR-335-5p was predicted to interact with IL21R, IL6 and CXCL2. Figure 9 shows the interactions among the 4 core genes and their target miRNAs.

## Discussion

Fibrotic diseases are generally caused by sustained damage to cells, which activates predisposing factors that lead to tissue injuries. Characterized by the chronic fibrosis of TA, PD is often associated with repeated trauma resulting from sexual activities or other vigorous exercises, which leads to penile malformation and corporo-venous occlusive erectile dysfunction (ED). This could cause psychological problems and have a significant impact on intimate relationships (Milenkovic et al., 2019a). To date, there are two dominant hypotheses for PD pathogenesis. For the first hypothesis, repeated trauma in the TA causes separation between the inner and outer layers, especially in the dorsomedial side between the cavernous bodies, which is formed by the septum (Graziottin, 2015). The second hypothesis suggests that minor trauma could separate the inner layer of TA from the sinusoidal tissue, resulting in the formation of micro hematoma in the connective tissue sleeve between TA and corpus cavernosum. Accordingly, the lesion was located on both the dorsomedial side of the TA and the distinctive extension into the corpus cavernosum (Milenkovic et al., 2019b). Disease enrichment analysis using Enrichr in this article showed that the top genes are enriched in various disorders. The first four disorders ranked as follows: research-related injuries, traumatic injury, injury wounds, wounds and injuries. This confirms the above two hypotheses.

KEGG pathway analysis indicated that the candidate genes were mainly involved in the regulation of the JAK-STAT signaling pathway and cytokine‒cytokine receptor interactions. The JAK-STAT signaling pathway plays critical roles in numerous biological processes, including immunity, cell division, cell death and tumor formation, although protein‒protein interactions (Shawky et al., 2022). Through this pathway, extracellular stimulations from chemical signals are transmitted to the cell nucleus to activate gene transcription. Janus kinases (JAKs), signal transducer and activator of transcription proteins (STATs), and receptors are the three key components in JAK-STAT signaling (Puigdevall et al., 2022). It has been reported that various diseases, including skin conditions, immune disorders and cancers, could be the consequence of dysregulated JAK-STAT signaling (Haftcheshmeh et al., 2022). Cytokines are small proteins or peptides secreted by specific types of cells that have been shown to be involved in the modulation of autocrine, paracrine and endocrine signaling (Dali-Youcef and Ricci, 2016; Yoshimura et al., 2018). Despite some similarities, the distinction between cytokines and hormones is still under investigation. Based on the above conclusions, the immune response is involved in PD fibrosis.

Studies have confirmed the presence of CD8^+^ cytotoxic T cells, B cells, and dendritic cells, as well as more specialized monocyte-derived CTL and MAIT cells in Peyronie’s plaques, which stimulate an inflammatory response (Milenkovic et al., 2021). The existence of these kinds of cells may suggest the involvement of the adaptive immune system in the progression of PD. In our article, immune cell infiltration analysis demonstrated that B cells, NK cells, T cells, macrophages, neutrophils and plasma cells are widely involved in PD fibrosis.

The significance of immune-related genes, especially their role in malignancies, has been partially demonstrated in previous urological studies. However, how immune-related genes are correlated with PD development is not well understood. In this article, we identified 37 hub genes with altered expression in PD using bioinformatics approaches. Then, 6 key immune-related genes were distinguished among the 37 hub genes: IL6, IL21R, IFNE, CXCL2, EGF, and ANGPTL5. Interestingly, the first five genes were the core genes in the PPI network. Proinflammatory IL-6 is a cytokine that induces acute-phase protein synthesis in response to trauma stimuli. Two different pathways are involved in IL-6 signaling: the classic signaling pathway and the trans-signaling pathway (Atar et al., 2017). The complex formed by IL-6, soluble IL-6 receptor and glycoprotein 130 (gp130) plays critical roles in the inflammatory pathways during the development of various autoimmune diseases, such as chronic inflammatory bowel disease, rheumatoid arthritis and asthma (Rose-John, 2020). Atar et al. (2017) proposed that the trans-signaling of IL-6 and the production of pentraxin 3 (PTX3) at the inflammation site could have contributed to PD pathogenesis. Although the remaining five genes have not been studied in PD, they are widely involved in the immune response. Further studies are still needed to elucidate the potential biological functions in PD pathogenesis for the candidate immune-related genes identified in this study.

The progression of PD is defined as the acute and chronic phases. In the acute phase, plaque is formed, which leads to penile pain, while in the chronic phase, the pain is usually mostly resolved with stabilized penile deformity. Currently, the pathophysiology of PD is not fully understood and remains the focus of many studies in this field (Ziegelmann et al., 2020).

The critical roles of miRNAs in many biological processes regulating development, differentiation, and organ function have been addressed previously (Saliminejad et al., 2019). Recently, miRNAs were reported to be involved in fibrosis development (Ghafouri-Fard et al., 2021). Dos Santos et al. (2022) found that the level of miR-29b was reduced in the fibrous plaque, TA and corpus cavernosum of PD patients compared with that in the control group. In this study, hsa-miR-335-5p was predicted to interact with three key immune-related genes: IL21R, IL6 and CXCL2. Another 67 miRNAs were predicted to play regulatory roles in the immune system of PD. In conclusion, miRNAs may serve as biomarkers in PD diagnosis.

There are still some limitations to our study. First, the quantity of the samples is relatively low. In addition, the dataset included in this study did not describe the phases of PD. The major limitation is that all the results were acquired through bioinformatics approaches. Second, the biological functions of the candidate genes in PD need to be further investigated since no related data have been published previously. For example, knocking out these genes in model animals may help elucidate their potential roles in regulating PD development.

## Conclusion

Taken together, the candidate hub genes and immune-related genes were identified through bioinformatic approaches, and functional studies of these genes elucidated the potential pathogenic mechanism of PD. The findings could help better elucidate how the immune system contributes to the progression of PD, thus shedding light on the development of new strategies to prevent and treat PD. Nevertheless, to assess the potential usage of these candidate genes in the clinic, more detailed studies are still needed to further investigate their regulatory mechanisms and related signaling pathways.

## Data Availability

The datasets presented in this study can be found in online repositories. The names of the repository/repositories and accession number(s) can be found in the article/supplementary material.
